# Biomolecular mechanisms of cardiac amyloidosis and its cardiovascular pathological basis

**DOI:** 10.3389/fimmu.2026.1832739

**Published:** 2026-05-29

**Authors:** Linlin Zhang, YaTing Jiao, Jia Guo

**Affiliations:** 1 First Clinical Medical College, Shanxi Medical University, Taiyuan, Shanxi, China; 2 Department of Cardiology, First Hospital of Shanxi Medical University, Taiyuan, Shanxi, China

**Keywords:** cardiac amyloidosis, cardiomyocyte toxicity, immunoglobulin light chain, protein misfolding, transthyretin

## Abstract

Cardiac amyloidosis (CA) is a progressive infiltrative cardiomyopathy most commonly caused by transthyretin (ATTR) or immunoglobulin light chain (AL) amyloid deposition in the myocardium, microvasculature, and conduction system, although rarer forms, including apolipoprotein A-I amyloidosis (AApoA-I) and serum amyloid A amyloidosis (AA), may also involve the heart. Although traditionally viewed as a disorder driven mainly by extracellular fibril accumulation, growing evidence indicates that myocardial injury in CA also reflects the effects of soluble toxic intermediates, proteostasis failure, immune-inflammatory activation, and secondary structural remodeling. In this review, we compare the major biomolecular mechanisms underlying cardiac injury in ATTR and AL, beginning with precursor destabilization, misfolding, and oligomer formation, and extending to direct cardiomyocyte toxicity, mitochondrial and metabolic stress, calcium dyshomeostasis, fibroinflammatory remodeling, extracellular matrix reorganization, microvascular dysfunction, and autonomic and electrophysiological abnormalities. We further emphasize that ATTR and AL, while sharing several downstream pathological consequences, differ in their dominant upstream drivers and in the relative contribution of deposition-dependent versus soluble toxicity-mediated injury. This integrated mechanistic framework helps explain disease heterogeneity, persistent dysfunction despite amyloid reduction, and the need for subtype-specific therapeutic strategies. A more precise understanding of these interconnected pathways may improve early diagnosis, risk stratification, and the development of therapies targeting both the initiating protein abnormality and the downstream mechanisms responsible for ongoing myocardial dysfunction.

## 1 Introduction

Cardiac amyloidosis (CA) is an infiltrative cardiomyopathy caused by the misfolding, aggregation, and tissue deposition of amyloidogenic precursor proteins within the myocardial interstitium, vascular walls, and conduction system (1, 2). Although CA was historically considered an uncommon cause of restrictive cardiomyopathy, the increasing use of bone scintigraphy, cardiac magnetic resonance, and molecular diagnostic testing has revealed that cardiac involvement is more prevalent than previously appreciated, particularly among older patients with heart failure with preserved ejection fraction and those with aortic stenosis (3–5). Clinically, CA is characterized by progressive ventricular wall thickening, impaired diastolic filling, conduction abnormalities, atrial arrhythmias, and eventually advanced heart failure (1, 2).

Among the systemic amyloidoses that involve the heart, transthyretin amyloidosis (ATTR) and immunoglobulin light chain amyloidosis (AL) are the two major subtypes with the greatest clinical relevance (1, 2). ATTR is driven by destabilization of the transthyretin tetramer, either in the setting of aging or disease-causing genetic variants, whereas AL results from the production of amyloidogenic monoclonal light chains by clonal plasma cells (2, 6, 7). Despite differences in precursor biology, both subtypes converge on a final common phenotype of myocardial stiffening, cardiomyocyte dysfunction, arrhythmia susceptibility, and progressive circulatory impairment (1, 2). However, the relative contribution of extracellular fibril deposition, soluble toxic intermediates, immune-inflammatory activation, and secondary tissue remodeling appears to differ between ATTR and AL (6–14).

Recent work has shifted the mechanistic understanding of CA beyond the traditional concept of passive amyloid infiltration. In addition to the space-occupying effects of fibrillar deposition, soluble misfolded monomers and oligomeric intermediates may exert direct proteotoxic effects on cardiomyocytes, disrupt mitochondrial function and calcium handling, alter extracellular matrix homeostasis, and impair microvascular and electrophysiological integrity (8–13). Emerging evidence also suggests that immune-inflammatory signaling and myocardial proteostasis stress are not merely secondary phenomena but may actively shape disease progression, tissue remodeling, and clinical heterogeneity (11, 14–16).

Several recent reviews have summarized the general pathophysiology, diagnosis, and treatment of cardiac amyloidosis (1, 2, 6, 7). In contrast, the present review focuses specifically on the biomolecular cascade linking precursor destabilization to myocardial injury, with emphasis on four aspects that remain insufficiently integrated in the existing literature. First, we compare the parallel but non-identical mechanisms of injury in ATTR and AL. Second, we highlight the contribution of soluble toxic intermediates in addition to mature fibrils. Third, we integrate immune-inflammatory signaling and proteostasis-related stress into the broader framework of cardiac remodeling. Fourth, we discuss the translational implications of these mechanisms for current and emerging therapeutic strategies. By organizing the evidence across these interconnected domains, this review aims to provide a more integrated mechanistic framework for understanding disease progression and identifying priorities for future research.

## 2 Basic overview

### 2.1 Major cardiac amyloid precursors and clinicopathological patterns

Amyloidosis comprises a group of protein misfolding disorders characterized by extracellular deposition of insoluble amyloid fibrils. Among the systemic forms that involve the heart, transthyretin amyloidosis (ATTR) and immunoglobulin light chain amyloidosis (AL) are the two major subtypes with the greatest clinical relevance (1, 2, 17). Although both may produce a final phenotype of ventricular wall thickening, diastolic dysfunction, arrhythmia susceptibility, and progressive heart failure, they differ substantially in precursor biology, disease tempo, and dominant mechanisms of myocardial injury (1, 2, 6, 17).

#### 2.1.1 ATTR type

ATTR is caused by misfolding and aggregation of transthyretin, a transport protein synthesized mainly by the liver (6, 17). Clinically, ATTR is classified into wild-type ATTR (ATTRwt) and variant ATTR (ATTRv) according to whether pathogenic mutations are present in the TTR gene (6, 17). ATTRwt occurs predominantly in older individuals(typically aged ≥70 years, with median ages of 75-84 years across studies), especially men(who account for 68%–94% of cases, with a male-to-female ratio of approximately 5-10:1), and is increasingly recognized as an underdiagnosed cause of heart failure with preserved ejection fraction and cardiac involvement in patients with aortic stenosis (3–5, 17). By contrast, ATTRv may present with variable combinations of cardiomyopathy and peripheral neuropathy, depending on the specific mutation and pattern of organ involvement (6, 17).

From a clinicopathological perspective, ATTR is often characterized by progressive extracellular amyloid deposition, ventricular wall thickening, restrictive filling physiology, impaired myocardial strain, and conduction abnormalities, while left ventricular ejection fraction may remain relatively preserved until later stages (1, 2, 6, 17). These features make ATTR an especially important model of chronic infiltrative and remodeling-driven cardiac injury.

#### 2.1.2 AL type

AL amyloidosis originates from the production of monoclonal immunoglobulin light chains by an abnormal plasma cell clone (7, 8). Compared with ATTR, AL often follows a more aggressive clinical course when the heart is involved, and cardiac dysfunction may progress rapidly even when the absolute burden of visible amyloid deposition is not extensive (18, 19). This pattern has led to the concept that, in AL, soluble light chain-related toxicity may contribute importantly to myocardial injury in addition to extracellular fibril accumulation (8–10).

Clinically, cardiac AL is often diagnosed late and is associated with marked elevation of cardiac biomarkers, particularly NT-proBNP and cardiac troponins, rapid deterioration in cardiac function, and poor prognosis if toxic light chain production is not promptly suppressed. Patient-survey and real-world data indicate substantial diagnostic delay, with more than one-third of patients remaining undiagnosed for at least 1 year after symptom onset and approximately 39%-47% already having advanced cardiac stage at diagnosis (18, 19). Thus, while both ATTR and AL are forms of cardiac amyloidosis, they should not be regarded as mechanistically interchangeable. ATTR more often reflects a chronic interplay between precursor instability and progressive tissue deposition, whereas AL more prominently combines fibril deposition with direct proteotoxic injury from circulating light chains (6–10, 17–19).

### 2.2 Why the heart is especially vulnerable: proteostasis sensitivity, mechanical demand, and limited regenerative reserve

The heart is particularly vulnerable to amyloid-related injury because it operates under continuous mechanical and metabolic demand while possessing only limited regenerative capacity (15, 16, 20, 21). Even under resting conditions, the myocardium is exposed to persistent workload and energetic stress, which become further amplified in the setting of pressure overload, ischemia, or neurohumoral activation (15, 16). In addition, cardiomyocytes are terminally differentiated cells with limited capacity for replacement after injury, and studies in humans and experimental models indicate that meaningful regenerative potential is highly restricted beyond early developmental stages (20, 21). As a result, the myocardium is especially sensitive to disturbances in protein homeostasis and to the cumulative effects of chronic proteotoxic stress (15, 16).

### 2.3 Literature search strategy and article selection

To provide an updated and mechanistically focused overview, we primarily searched PubMed and Web of Science for English-language articles published over the past decade, with particular emphasis on studies published between 2020 and 2025. Key search terms included combinations of “cardiac amyloidosis,” “transthyretin,” “light chain,” “proteotoxicity,” “inflammation,” and “microvascular dysfunction.” Priority was given to primary mechanistic studies, translational investigations, human tissue-based analyses, and representative clinical reports relevant to ATTR and AL cardiac amyloidosis. Selected review articles were used mainly to support broader conceptual framing, disease overview, and contextual interpretation of the primary literature.

## 3 Pathological mechanisms of cardiac injury in cardiac amyloidosis

### 3.1 Precursor destabilization, toxic intermediates, and fibril formation in ATTR and AL

Amyloid formation in cardiac amyloidosis begins with instability of the precursor protein rather than with fibril deposition alone. In both ATTR and AL, the upstream pathogenic process involves loss of native conformational stability, generation of misfolded intermediates, and eventual assembly into β-sheet-rich aggregates (6, 7, 17, 22, 23). However, the molecular drivers of this process differ substantially between the two major subtypes. Overall, evidence is strongest for the role of precursor destabilization and partial unfolding, whereas the precise contribution of specific oligomeric states and tissue-selective aggregation remains incompletely defined in human disease (7, 22–24).

#### 3.1.1 ATTR type

Under physiological conditions, transthyretin circulates predominantly as a stable tetramer (17, 25). ATTR amyloidosis is initiated when the tetramer dissociates into monomers, which are more prone to misfolding and entry into the amyloidogenic pathway (17, 25). In ATTRv, pathogenic point mutations reduce tetramer stability and alter folding kinetics, oligomer dynamics, and susceptibility to proteolytic processing, thereby increasing the tendency toward amyloid formation (22, 25). By contrast, certain variants, such as T119M, have been associated with relative stabilization of the TTR tetramer, although their protective effect appears to vary across populations and study settings (26, 27).

In ATTRwt, the mechanism of age-related destabilization is less completely understood. Current evidence suggests that age-related oxidative modifications of TTR, particularly methionine/cysteine oxidation and carbonylation, may reduce TTR thermodynamic stability and increase amyloidogenic potential, thereby contributing to the late-onset predominance of wild-type disease (17, 28). Thus, in ATTR, precursor destabilization is a well-established initiating event, whereas the precise factors that govern tissue selectivity, disease tempo, and the relative toxicity of different soluble intermediates remain areas of ongoing investigation (17, 22, 25, 28).

#### 3.1.2 AL type

In AL amyloidosis, the precursor protein is an immunoglobulin light chain secreted by a clonal plasma cell population (7, 29). A defining feature of AL is the marked sequence heterogeneity of the light chain variable domain, which arises from somatic hypermutation and can profoundly alter folding stability and amyloidogenicity (29, 30). Importantly, not all monoclonal light chains are amyloidogenic. Amyloid formation appears to depend on whether sequence variation destabilizes the native fold sufficiently to permit partial unfolding, exposure of aggregation-prone regions, and formation of misfolded intermediates (29, 30).

Proteolysis and conformational dynamics may further accelerate this process. AL deposits often contain light chain fragments, suggesting that proteolytic cleavage is not merely a passive consequence of deposition but may participate in fibril formation itself (31, 32). In particular, truncation of the constant domain can enhance fibrillogenicity, while alternative conformational and dimeric states may influence tissue-specific proteolysis and aggregation behavior (31, 32). Compared with ATTR, the upstream mechanism in AL is therefore more sequence-specific and structurally heterogeneous. Although biochemical and structural studies strongly support this model, the exact determinants of organ tropism and the relative contribution of soluble intermediates versus deposited fibrils remain incompletely resolved in patients (7, 29–32).

### 3.2 Proteostasis failure and protein quality control stress

Protein quality control (PQC) is essential for long-term cardiomyocyte integrity. In the healthy myocardium, molecular chaperones, the ubiquitin-proteasome system, and the autophagy-lysosome pathway cooperate to recognize, refold, or eliminate damaged and misfolded proteins (15, 16). When this network is overwhelmed or dysregulated, abnormal proteins and aggregates accumulate, leading to proteotoxic stress, mitochondrial dysfunction, and impaired cellular homeostasis (15, 16, 35, 36). These uncleared misfolded species and damaged cellular components may function as damage-associated molecular patterns (DAMPs), thereby promoting innate immune signaling and maladaptive remodeling, although in cardiac amyloidosis this link remains more strongly supported by indirect and extrapolative evidence than by direct causal demonstration in human tissue (33, 34).

In cardiac amyloidosis, sustained precursor misfolding imposes chronic stress on myocardial proteostasis. In ATTR, destabilized transthyretin monomers primarily generate extracellular amyloid deposition, but misfolded intermediates may also contribute to ongoing cellular stress and maladaptive remodeling (6, 17, 37). In AL, circulating amyloidogenic light chains and their soluble oligomers appear to exert a more direct toxic effect on cardiomyocytes, interfering with mitochondrial function, intracellular quality control systems, and stress-response pathways (8–10, 35, 36). Although the relative contribution of these mechanisms likely differs between ATTR and AL, both subtypes ultimately challenge the ability of the myocardium to maintain protein homeostasis under persistent stress (2, 6, 17, 35–37).

From a translational perspective, this proteostasis-centered view is clinically relevant because it links precursor instability to downstream cellular injury and helps explain why reducing amyloid burden alone may not fully reverse established myocardial dysfunction. It also provides a mechanistic rationale for therapeutic strategies aimed at stabilizing precursor proteins, reducing toxic precursor production, or modulating downstream stress-response pathways (15, 16).

### 3.3 Direct cardiomyocyte toxicity: oxidative stress, mitochondrial injury, and calcium dyshomeostasis

Direct cardiomyocyte toxicity is a central component of cardiac amyloidosis and extends beyond the passive mechanical effects of extracellular amyloid deposition. In both ATTR and AL, toxic precursor species and their soluble intermediates appear capable of inducing intracellular stress responses that impair myocardial function before or in parallel with extensive fibril accumulation. Among the downstream consequences, oxidative stress, mitochondrial dysfunction, metabolic disturbance, and abnormal calcium handling form an interconnected pathogenic axis that contributes to impaired relaxation, contractile dysfunction, and progressive cardiac remodeling (2, 6, 10, 35–40).

In AL cardiac amyloidosis, soluble immunoglobulin light chains are thought to exert particularly strong direct toxic effects on cardiomyocytes. Experimental studies have shown that amyloidogenic light chains can rapidly induce diastolic dysfunction, increase intracellular reactive oxygen species, reduce mitochondrial membrane potential, disrupt metabolic homeostasis, and promote apoptosis (8, 9, 38, 39, 41, 42). Proteomic and functional studies further support the involvement of mitochondrial networks in AL-related cardiotoxicity. In particular, cardiotoxic light chains have been associated with alterations in proteins related to ATP synthesis and oxidative phosphorylation, fatty acid metabolism, outer mitochondrial membrane transport, and cristae organization, including ATP synthase subunits, HADHB, VDAC1, and MIC60/IMMT. These findings suggest that mitochondrial injury is not merely a secondary consequence but part of the direct proteotoxic response to circulating light chains (35, 36, 38, 42). In parallel, evidence from human cardiac spheroid and cardiomyocyte models indicates that amyloidogenic light chains can disturb calcium transients and impair excitation-contraction coupling, accompanied by abnormalities in calcium-handling proteins such as SERCA2a (10). Mechanistically, AL light chain toxicity may involve oxidative stress and non-canonical stress signaling, particularly via the TAB1-p38α axis, which may promote mitochondrial injury and downstream cardiomyocyte dysfunction, while mediators such as stanniocalcin-1, a stress-responsive glycoprotein implicated in calcium homeostasis, may further disrupt calcium handling and impair contractility (9, 43). Taken together, current evidence suggests that AL is characterized by a relatively prominent soluble proteotoxic component, although most mechanistic support still derives from *in vitro* and experimental systems rather than direct causal demonstration in human myocardium (8–10, 38, 39, 41–43).

In ATTR cardiomyopathy, cardiomyocyte injury appears to arise from the combined effects of chronic extracellular deposition and the toxicity of misfolded transthyretin species. In addition to increasing myocardial stiffness, misfolded TTR monomers and oligomers may induce oxidative stress, mitochondrial stress, and energetic inefficiency, thereby contributing to cellular dysfunction even before extensive mature fibril deposition is established (6, 17, 37, 40). Experimental studies have shown that TTR aggregates can increase reactive oxygen species, disrupt myofilament organization, and delay calcium handling (13, 37). In deposition-based models, cardiomyocytes cultured on TTR fibril-coated substrates display slower contraction-relaxation kinetics, prolonged calcium transients, reduced contractile force, and decreased connexin-43 expression, suggesting that extracellular deposition can secondarily disturb electrical coupling and excitation–contraction coordination (37). Additional electrophysiological studies also support the view that TTR oligomers and fibrils may alter calcium cycling and membrane electrical properties, thereby increasing arrhythmogenic susceptibility (13). Overall, in ATTR, calcium-handling abnormalities and contractile dysfunction likely reflect both direct proteotoxic stress from soluble intermediates and the progressive structural remodeling caused by extracellular amyloid deposition (6, 13, 17, 37, 40).

**Figure 1:**
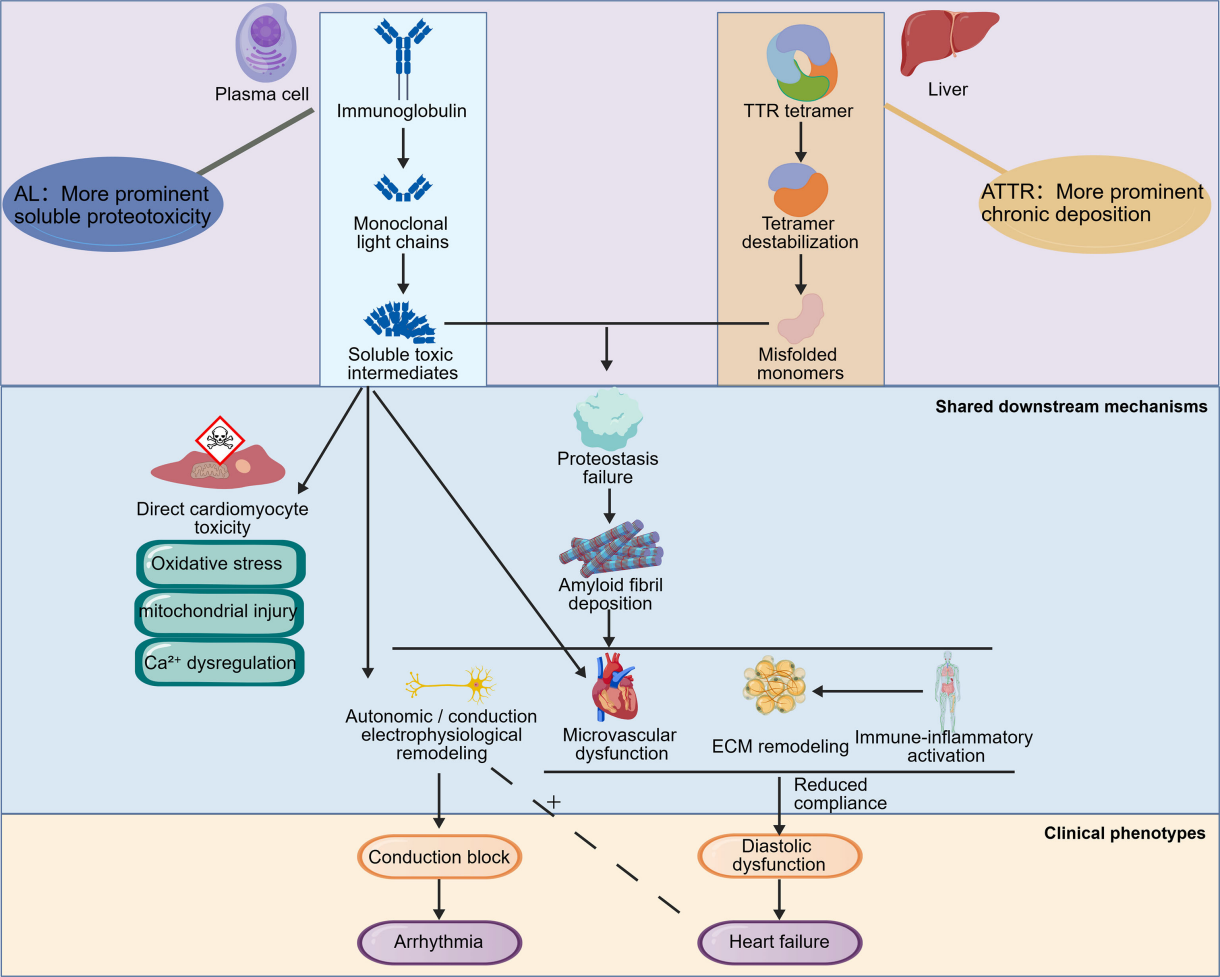
Integrated biomolecular mechanisms of cardiac injury in ATTR and AL cardiac amyloidosis (61).

From a translational perspective, this integrated view is important because it highlights that myocardial dysfunction in cardiac amyloidosis cannot be explained solely by fibril burden. Instead, subtype-specific differences in direct cardiomyocyte toxicity may influence disease tempo, reversibility, and therapeutic responsiveness. In particular, AL appears more strongly linked to rapid light chain-mediated cellular injury, whereas ATTR more often reflects a sustained interaction between soluble proteotoxic stress and chronic deposition-related remodeling (10, 38–40). A schematic comparison of these subtype-specific injury patterns is shown in **Figure 1**.

### 3.4 Immune-inflammatory activation and fibroinflammatory remodeling

Emerging evidence suggests that immune-inflammatory activation is not merely a secondary bystander in cardiac amyloidosis but may participate in the amplification of myocardial injury and remodeling (14, 44, 46). Building on precursor misfolding, proteotoxic stress, and impaired protein quality control, persistent misfolded protein species and damaged cellular components may promote inflammatory signaling within the myocardial microenvironment (2, 33, 34, 46). These uncleared stress-associated molecules may function as DAMPs, thereby engaging innate immune signaling pathways and further amplifying myocardial inflammation and remodeling (33, 34). At present, however, the strength of evidence is not uniform across all pathways, and many mechanistic links remain better supported by experimental and translational observations than by direct causal proof in human tissue (2, 44, 46).

In ATTR cardiomyopathy, recent human studies have suggested that myocardial inflammation may accompany transthyretin deposition and may carry potential prognostic relevance (14, 44). This observation supports the concept that amyloid deposition, cellular stress, and inflammatory remodeling may interact in a mutually reinforcing manner rather than representing strictly separate processes (14, 44, 46). In AL amyloidosis, the inflammatory component may be more closely coupled to the direct toxic effects of circulating light chains and to downstream cellular stress responses, although the precise inflammatory hierarchy remains less clearly defined (18, 35, 36, 46). Thus, while ATTR and AL differ in their dominant upstream triggers, both may converge on a myocardial milieu in which inflammatory signaling contributes to tissue injury and disease progression (14, 18, 44, 46).

Inflammation is also closely linked to fibroinflammatory remodeling. In cardiac amyloidosis, extracellular matrix turnover is not simply a passive response to amyloid deposition (11, 45, 46). Changes in matrix metalloproteinases, tissue inhibitors of metalloproteinases, and extracellular matrix organization suggest that inflammatory and proteolytic remodeling pathways may actively shape myocardial stiffness, interstitial expansion, and structural disarray (45, 46). From a translational perspective, this immune-inflammatory and fibroremodeling framework is clinically relevant because it implies that effective treatment may require not only reduction of toxic precursor proteins or amyloid burden, but also attention to downstream inflammatory and matrix-mediated injury, particularly in more advanced stages of disease (2, 11, 14, 45, 46).

### 3.5 ECM structural changes and mechanisms of decreased myocardial compliance

Extracellular matrix and interstitial remodeling provide the principal structural basis for reduced ventricular compliance in cardiac amyloidosis. Human biopsy, imaging, and clinicopathological studies indicate that one of the core structural abnormalities in CA is expansion of the extracellular compartment, which reflects both direct amyloid deposition and accompanying fibrotic remodeling (2, 17, 45, 47). As the interstitium enlarges and stiffens, higher filling pressures are required to achieve limited diastolic volume increase, thereby promoting restrictive physiology and impaired ventricular compliance (2, 17, 45, 47).

These structural abnormalities are not explained solely by passive amyloid accumulation. In addition to the space-occupying effects of fibrils, altered matrix turnover and abnormal extracellular remodeling may further increase passive stiffness and sustain myocardial disorganization (11, 45, 46). Although the broader inflammatory and proteolytic remodeling framework has been discussed above, studies showing altered matrix metalloproteinase and tissue inhibitor profiles support the view that extracellular remodeling is an active component of disease progression rather than a purely static consequence of deposition (11, 45, 48). From a structural perspective, these matrix changes may alter collagen organization, crosslinking, and interstitial continuity, thereby further compromising myocardial compliance (11, 45, 46, 48).

ECM abnormalities may also influence myocardial function by disturbing cell–matrix and cell–cell interactions that are required for coordinated force transmission and tissue integrity. Experimental deposition models have shown that transthyretin-related interstitial changes can disrupt adhesion complexes and contribute to altered sarcomeric architecture, disturbed calcium transients, and reduced contractile force (37). Thus, in cardiac amyloidosis, extracellular remodeling should be viewed not only as a marker of tissue infiltration, but also as a structural mediator through which protein deposition is translated into ventricular stiffness, impaired relaxation, and progressive mechanical dysfunction (37, 40, 45–48).

### 3.6 Microvascular dysfunction and ischemic stress

In cardiac amyloidosis, myocardial ischemic stress is usually not caused by epicardial coronary atherosclerosis, but rather by abnormalities of the coronary microcirculation. Pathological studies show that amyloid deposition commonly involves the intramural coronary microvasculature and the surrounding interstitium, leading to luminal narrowing, decreased vascular compliance, and increased microcirculatory resistance (12, 45, 50). This pattern reinforces the concept that microvascular dysfunction is an integral component of cardiac injury in CA rather than a coincidental finding. Clinical imaging studies further corroborate this view. Positron emission tomography (PET) and cardiac magnetic resonance (CMR) have shown that CA patients may exhibit impaired myocardial blood flow reserve even in the absence of significant epicardial coronary stenosis, and this abnormality appears to correlate with ventricular wall thickening and amyloid burden (12, 50, 51). Thus, current human evidence supports coronary microvascular dysfunction as a clinically relevant mechanism of non-obstructive ischemia in CA, although the precise contribution of different vascular and interstitial factors may vary across disease stages and subtypes.

From a pathophysiological perspective, sustained microvascular insufficiency may keep the myocardium in a state of relative ischemia and energetic stress, thereby interacting with oxidative stress, mitochondrial dysfunction, and extracellular remodeling to further impair cardiac performance (12, 49, 51). The relative contribution and dominant mechanisms of this process may differ between ATTR and AL. In ATTR cardiomyopathy, progressive interstitial expansion, tissue stiffening, and perivascular amyloid infiltration may contribute to reduced perfusion reserve and increased microvascular resistance (45, 50, 51). In AL amyloidosis, in addition to deposition-related changes, circulating light chains may also contribute to endothelial and microvascular dysfunction; *ex vivo* studies using human microvessels have demonstrated impaired vascular reactivity and apoptotic injury after exposure to amyloidogenic light chains (49). Overall, microvascular dysfunction should be viewed as a key intermediary linking amyloid deposition, metabolic stress, and progressive myocardial dysfunction (12, 49–51). Key mechanistic differences between ATTR and AL cardiac amyloidosis are summarized in **Table 1**.

**Table 1:** Key mechanistic comparison between ATTR and AL cardiac amyloidosis.

Domain	ATTR	AL
Precursor source	Liver-derived transthyretin (TTR)	Monoclonal light chains from clonal plasma cells
Primary initiating mechanism	Tetramer destabilization, monomer misfolding, oxidative/post-translational modification	Sequence heterogeneity, partial unfolding, proteolysis, conformational instability
Predominant toxic species	Soluble intermediates plus chronic fibrillar deposition; more prominent deposition-driven remodeling	Soluble toxic light chains/oligomers plus fibrillar deposition; more prominent soluble proteotoxicity
Main pattern of myocardial injury	Chronic infiltration, interstitial expansion, reduced compliance, and structural remodeling	More prominent direct cardiomyocyte toxicity and rapid functional decline
Shared downstream mechanisms	Proteostasis failure, immune-inflammatory activation, ECM remodeling, microvascular dysfunction, and electrophysiological remodeling	
Clinical tempo	Usually slower, progressive, and remodeling-dominant	Often faster and more severe once cardiac involvement occurs
Main therapeutic priority	TTR stabilization and/or reduction of precursor production	Rapid suppression of toxic light chain production by clone-directed therapy
Emerging shared targets	Proteostasis-related stress, immune-inflammatory remodeling, ECM/microvascular injury, and amyloid clearance	

### 3.7 Autonomic, electrophysiological, and conduction remodeling

Electrical instability in cardiac amyloidosis is not solely explained by ventricular stiffening or chamber remodeling. Instead, autonomic dysfunction, direct involvement of the conduction system, and electrophysiological remodeling of the working myocardium together contribute to the high burden of arrhythmias and conduction disorders observed in CA (17, 55, 56). These abnormalities can manifest clinically as orthostatic intolerance, reduced exercise tolerance, atrial arrhythmias, progressive atrioventricular conduction delay, bundle branch block, and high-grade conduction disease (17, 55, 56).

Autonomic involvement appears particularly relevant in hereditary transthyretin amyloidosis (ATTRv). Clinical studies have shown that patients may exhibit orthostatic hypotension, impaired heart rate variability, and evidence of sympathetic denervation, with ^123I-MIBG imaging showing decreased myocardial sympathetic uptake and increased washout rate in some ATTR patients, potentially even before overt structural cardiac involvement becomes apparent (52–54). These findings suggest that neurocardiac dysfunction is not merely an epiphenomenon, but may contribute to symptom burden, exercise intolerance, and risk stratification in selected CA populations (52–54).

At the tissue level, conduction abnormalities in CA reflect both structural and functional mechanisms. Amyloid infiltration of the sinoatrial node, atrioventricular node, and His-Purkinje system, together with focal fibrosis and interstitial remodeling, can impair continuity of the conduction pathways and progressively reduce conduction safety (55). This provides a structural basis for PR prolongation, widening of the QRS complex, bundle branch block, and advanced atrioventricular block (55).

In parallel, the working myocardium may undergo electrophysiological remodeling. Gap junction organization, particularly involving connexin-43, may be disrupted by extracellular expansion, altered intercalated disc microdomains, and chronic stress-related remodeling, thereby slowing conduction velocity and increasing anisotropy (13, 37). Experimental studies further suggest that transthyretin-related deposition and aggregate toxicity can disturb calcium handling, adhesion complexes, and electrophysiological stability (13, 37). Similarly, in AL amyloidosis, circulating light chains may contribute to electrophysiological vulnerability through direct proteotoxic effects on calcium handling and oxidative stress pathways, potentially increasing arrhythmogenic susceptibility even in the absence of extensive fibril deposition (8–10).

Overall, current evidence supports the view that autonomic dysfunction, conduction system infiltration, and electrophysiological remodeling of the working myocardium are complementary rather than competing mechanisms. From a translational perspective, these interconnected abnormalities may partly explain why conventional antiarrhythmic strategies are often challenging in CA, and why close rhythm surveillance and device-based management assume particular importance in selected patients (55, 56).

### 3.8 Translational implications: from mechanism to therapy

The mechanistic framework of cardiac amyloidosis has important translational implications because it helps explain why treatment cannot rely solely on the recognition of myocardial infiltration, but must also address upstream protein instability and downstream tissue injury. In both ATTR and AL, early intervention is likely to be more effective before extensive structural remodeling, microvascular dysfunction, and electrophysiological derangement become established. Thus, a mechanism-based view of CA supports both earlier diagnosis and subtype-specific therapeutic strategies (2, 17–19, 57–60).

In ATTR cardiomyopathy, the central role of transthyretin destabilization provides a clear rationale for therapies that stabilize the TTR tetramer or reduce the production of amyloidogenic precursor protein. Tafamidis represents the best-established example of a mechanism-directed therapy based on TTR stabilization (57). More broadly, the biological model of ATTR also suggests that treatment response may depend not only on reducing ongoing amyloid formation but also on the extent of pre-existing myocardial remodeling, including fibrosis, microvascular impairment, and conduction system involvement (17, 57, 60). This helps explain why therapeutic benefit is often greatest when intervention occurs before advanced cardiac damage becomes irreversible.

In AL amyloidosis, the translational priority is even more tightly linked to the rapid suppression of toxic light chain production, because circulating amyloidogenic light chains may directly injure cardiomyocytes before or in parallel with substantial fibril deposition. This mechanism helps explain why hematologic response is so closely tied to cardiac outcomes in AL (18, 19, 58). At the same time, current mechanistic evidence suggests that reducing precursor burden alone may not fully reverse downstream consequences such as mitochondrial injury, extracellular remodeling, or electrical instability once these processes are established (18, 19, 58). Therefore, although clone-directed therapy remains the cornerstone of AL treatment, the disease biology also supports adjunctive strategies aimed at limiting proteotoxic stress and secondary tissue injury.

A broader translational lesson from both ATTR and AL is that several downstream pathways may eventually become therapeutically relevant, even if they are not yet fully validated clinical targets. Another potentially important direction is the development of monoclonal antibodies targeting amyloid fibrils, with the aim of promoting immune-mediated clearance of deposited amyloid, including macrophage-associated phagocytic mechanisms (59). Although this strategy remains under active investigation, it illustrates how immunological mechanisms may be harnessed therapeutically in CA (59). Alongside proteostasis-related stress, matrix remodeling, microvascular dysfunction, and arrhythmogenic remodeling, most of these downstream mechanisms remain investigational (2, 17–19, 59). Nevertheless, integrating these pathways into the conceptual model of CA is clinically valuable because it helps explain persistent dysfunction despite reduction of amyloid burden and highlights priorities for future therapeutic development (2, 17–19, 57–60).

## 4 Conclusion and future perspectives

Cardiac amyloidosis is increasingly recognized as a multifaceted cardiomyopathy in which myocardial injury cannot be explained solely by passive amyloid deposition. Instead, disease progression reflects the convergence of precursor instability, soluble proteotoxic intermediates, proteostasis failure, immune-inflammatory activation, extracellular remodeling, microvascular dysfunction, and electrophysiological derangement. Although ATTR and AL share several downstream pathological consequences, they differ in their dominant upstream drivers and in the relative contribution of deposition-dependent versus soluble toxicity-mediated injury. In this context, ATTR more often reflects a chronic interplay between transthyretin destabilization, extracellular accumulation, and progressive tissue remodeling, whereas AL is more prominently shaped by the direct cardiotoxic effects of circulating amyloidogenic light chains in addition to fibril deposition.

This integrated framework has important clinical implications. First, it helps explain why early diagnosis and timely intervention are critical, since many downstream changes—including fibrosis, impaired microvascular reserve, and conduction system remodeling—may become only partially reversible once established. Second, it supports a subtype-specific understanding of therapy, in which precursor-directed interventions remain central, but may not be sufficient to fully restore myocardial function after secondary injury pathways have developed. Third, it highlights why persistent symptoms or functional impairment may continue despite reduction of amyloid burden, particularly in patients with advanced structural and electrophysiological remodeling.

Several questions remain insufficiently resolved and should be prioritized in future research. These include the precise *in vivo* role of soluble oligomeric intermediates, the degree to which immune-inflammatory mechanisms actively drive disease progression in different CA subtypes, the key nodes of proteostasis failure in the stressed myocardium, and the relative reversibility of matrix, microvascular, and electrical remodeling after upstream disease control. Greater integration of human tissue studies, translational model systems, and subtype-specific clinical phenotyping will be essential to refine these issues. Ultimately, a more precise mechanistic understanding of CA may support not only earlier diagnosis and better risk stratification, but also the development of therapies that target both the initiating protein abnormality and the downstream pathways responsible for persistent myocardial dysfunction.
